# Effect of Angiotensin–Neprilysin Versus Renin–Angiotensin System Inhibition on Renal Outcomes: A Systematic Review and Meta-Analysis

**DOI:** 10.3389/fphar.2021.604017

**Published:** 2021-11-19

**Authors:** Ying Xu, Yang Chen, Jia Wei Zhao, Chao Li, Amanda Y Wang

**Affiliations:** ^1^ The Kidney Disease Center, The First Affiliated Hospital, College of Medicine, Zhejiang University, Hangzhou, China; ^2^ The Renal and Metabolic Division, The George Institute for Global Health, University of New South Wales, Newtown, NSW, Australia; ^3^ The Third Grade Laboratory Under the National State, Administration of Traditional Chinese Medicine, Hangzhou, China; ^4^ Key Laboratory of Kidney Disease Prevention and Control Technology, Hangzhou, China; ^5^ National Key Clinical Department of Kidney Diseases, Institute of Nephrology, Zhejiang University, Hangzhou, China; ^6^ The Faculty of Medicine, Bond University, Gold Coast, QLD, Australia; ^7^ Cardiovascular Center, Beijing Tongren Hospital, Capital Medical University, Beijing, China; ^8^ The Department of Renal Medicine, Concord Repatriation General Hospital, Concord, NSW, Australia; ^9^ Concord Clinical School, The University of Sydney, Sydney, NSW, Australia

**Keywords:** sacubitril/valsartan, renin–angiotensin–aldosterone system, renal outcomes, systematic review, meta-analysis

## Abstract

**Aims:** We aim to perform a systematic review and meta-analysis examining randomized controlled trials assessing the efficacy and safety of sacubitril/valsartan in patients on renal outcomes, in comparison with the renin–angiotensin–aldosterone system inhibitor (RAASi).

**Methods:** Eligible studies were retrieved on MEDLINE, EMBASE, and Cochrane until September 2021. The primary outcome was the incidence of renal impairment, which was defined as the composite of increases in serum creatinine by >0.3 mg/dl and/or a reduction in eGFR ≥25%, development of ESRD, or renal death. We pooled relative risks (RRs) with 95% confidence intervals (CIs) or the mean difference with 95% CIs for the variables.

**Results:** Our search yielded 10 randomized controlled trials with a total of 18,362 patients. Compared with RAASi treatment, patients treated with sacubitril/valsartan had lower incidence of composite renal impairment (10 studies, 18,362 patients, RR 0.84; 95% CI 0.72–0.96, *p* = 0.01; *I* ^*2*^ = 22%), ESRD development (3 studies, 13,609 patients, RR 0.53; 95% CI 0.30–0.96, *p* = 0.03; *I* ^*2*^ = 0%), drug discontinuation due to renal events (4 studies, 9,995 patients, RR 0.58; 95% CI 0.40–0.83, *p* = 0.003; *I* ^*2*^ = 47%), severe hyperkalemia (6 studies, 16,653 patients, RR 0.80; 95% CI 0.68–0.93, *p* = 0.01; *I* ^*2*^ = 25%) and a slower eGFR decline (4 studies, 13,608 patients, WMD 0.56; 95% CI 0.36–0.76, *p* < 0.00001; *I* ^*2*^ = 65%). Subgroup analysis demonstrated that sacubitril/valsartan was associated with a lower incidence of renal impairment in patients with heart failure and preserved ejection fraction (HFpEF), but not in those with heart failure and reduced ejection fraction (HFrEF). The superior renal function preservation of sacubitril/valsartan treatment was not associated with different baseline eGFR levels and follow-up duration. There was a smaller increase in the change in the urine albumin-to-creatinine ratio (UACR) (3 studies, 9,114 patients, SMD 0.06; 95% CI 0.02–0.10, *p* = 0.003; *I* ^*2*^ = 14%) with sacubitril/valsartan treatment. However, patients with heart failure appeared to have increased microalbuminuria, not patients without HF (*p* = 0.80 for interaction).

**Conclusion:** Sacubitril/valsartan was associated with a lower incidence of composite renal impairment especially in patients with HFpEF, but higher microalbuminuria in patients with heart failure (both HFrEF and HFpEF) compared with RAASi. The lower incidence of severe hyperkalemia and drug discontinuation due to renal events in patients with sacubitril/valsartan treatment demonstrated its superior safety compared with RAASi.

## Introduction

Cardiovascular disease is a leading cause of death globally and is associated with poor patient-centered outcomes and high economic burdens (Savarese and Lund, 2017). Angiotensin receptor neprilysin inhibitors (ARNi) is a new class of drugs with neprilysin inhibition and angiotensin II receptor type 1 (AT1) blockade. It has shown an improvement in ventricular function by counteracting the detrimental effects of renin–angiotensin–aldosterone system (RAAS) activation and blocking neprilysin to degrade endogenous natriuretic peptides, and has demonstrated benefits in patients with mild-to-moderate arterial hypertension and heart failure (Mangiafico et al., 2013). Sacubitril/valsartan, also known as LCZ696 or Entresto, is the first agent of angiotensin receptor neprilysin inhibitor to be approved for use in this setting (Gu et al., 2010).

Recent trials have revealed the beneficial effect of sacubitril/valsartan on hypertension and heart failure with reduced ejection fraction (HFrEF) and preserved ejection fraction (HFpEF) (Wang et al., 2019). Meanwhile, the vicious circle of chronic kidney disease (CKD) and cardiovascular disease (CVD) (Hein et al., 2019) raised the assumption that sacubitril/valsartan could protect kidney function by ameliorating heart failure. However, the effect of sacubitril/valsartan on renal function remains unclear. In the PARADIGM-HF (McMurray et al., 2014) trial and its secondary analysis (Damman et al., 2018), the use of sacubitril/valsartan was associated with an improvement in both cardiovascular and renal outcomes, compared with valsartan. However, the United Kingdom HARP-III (Haynes et al., 2018) study did not show differences in cardiovascular mortality and renal function between sacubitril/valsartan and valsartan groups.

This systematic review aimed to assess the current evidence from randomized trials of sacubitril/valsartan on renal efficacy and safety for patients with and without heart failure.

## Materials and Methods

### Search Strategy and Study Selection

Medline, EMBASE, and the Cochrane Register were searched till September 2021 by two authors (YX and YC) independently using search terms including “neprilysin inhibitor/angiotensin II-receptor blocker” and relevant terms, the names of ARNi drugs, and terms related to randomized clinical trials. Details of the search strategy for the three databases are provided in Supplementary Appendix Table S1.

**FIGURE 1:**
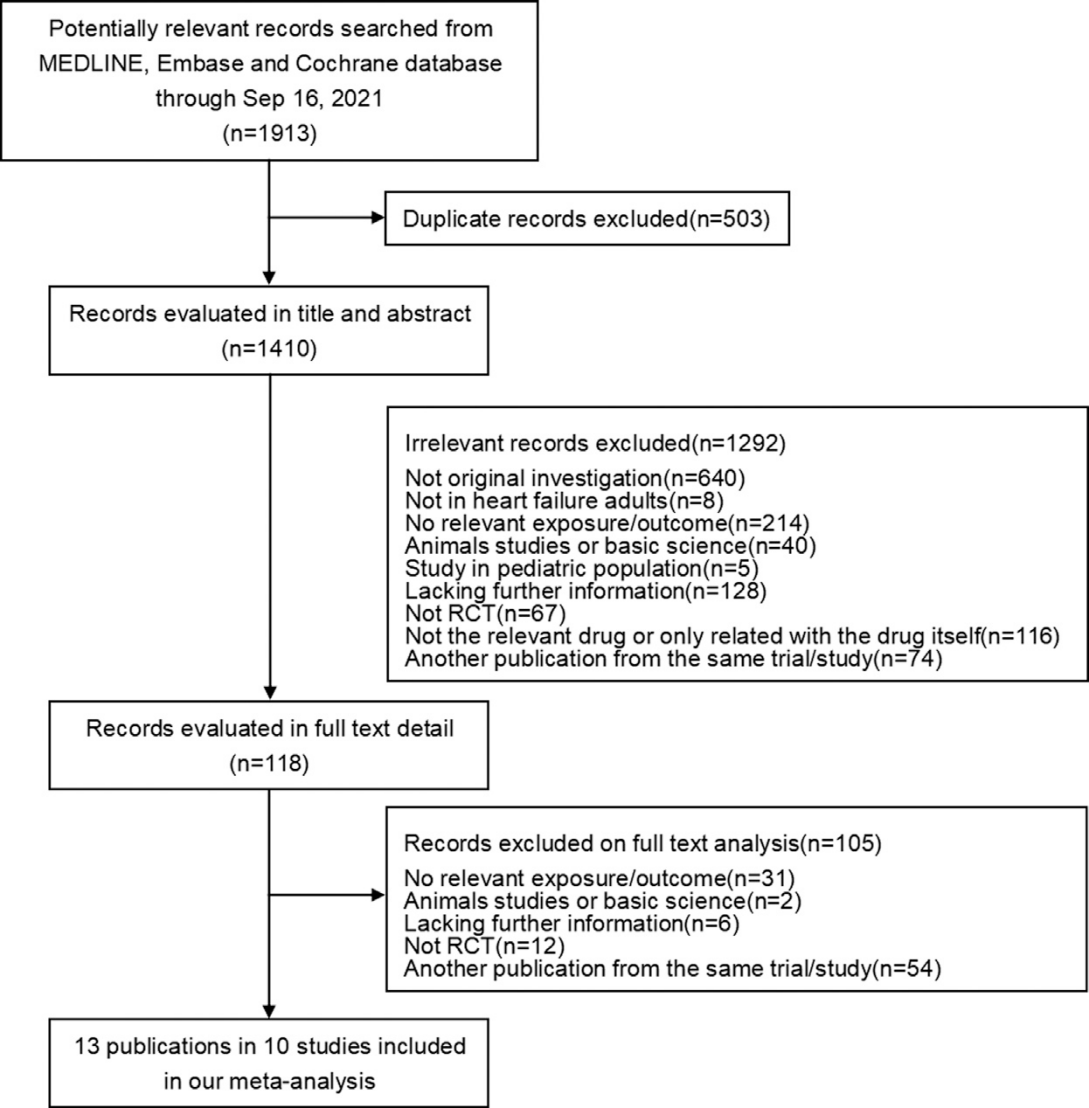
Screening flow chart.

Eligible studies had to meet the following inclusion criteria: 1) randomized controlled trials, quasi-randomized controlled trials or cross-over trials; 2) Study participants were adult patients; 3) Oral sacubitril/valsartan was used in the intervention group, while angiotensin-converting enzyme inhibitors or angiotensin II-receptor blockers alone were used in the control group; and 4) availability of renal outcomes. Study selection was independently completed by two reviewers (YX and YC). Any disagreements on the eligibility of a study were resolved by discussions with a third reviewer (AW). The literature screen flow chart is shown in Figure 1.

### Study Outcomes

The primary outcome was the incidence of renal impairment, which was defined as a composite of increases in serum creatinine by >0.3 mg/dl and/or a reduction in estimated glomerular filtration rate (eGFR) ≥25%, development of end-stage renal disease (ESRD), or renal death. The secondary outcomes included 1) a change in eGFR level during the follow-up period; 2) a change of urine albuminuria-to-creatinine ratio (UACR) level during the follow-up period; 3) adverse events (e.g., incidence of development of acute kidney injury, symptomatic hypotension, hyperkalemia, angioedema, and cough); and 4) compliance with the trial medications (trial drug completion, target dose achievement, drug discontinuation due to any adverse events, or renal events).

### Data Extraction and Quality Assessment

Data extraction was carried out independently by two authors (YX and YC) using standard data extraction forms. Data extracted from each study included baseline characteristics of study participants, drug use, follow-up duration, primary and secondary outcomes. The risk of bias associated with each included trial was assessed using the Cochrane risk of bias criteria.

### Data Synthesis and Analysis

Random effect models were used to pool relative risks with 95% CIs for individual studies when heterogeneity existed between studies; otherwise, fixed effect models were used. Weighted mean difference or standard mean difference were used to analyze the continuous variables according to the distribution of the values. The heterogeneity between studies was assessed through the calculation of *I* ^*2*^ values and corresponding *p*-values, with *I* ^*2*^ ≥ 50% or *p* < 0.05 denoting heterogeneity. Prespecified subgroup analysis was performed to explore sources of heterogeneity according to the following characteristics: the presence of baseline cardiovascular disease and type of heart failure, i.e., with heart failure and without heart failure, heart failure with reduced ejection fraction (HFrEF), and heart failure with preserved ejection fraction (HFpEF); the baseline eGFR level, i.e., eGFR <60 ml/min/1.73 m^2^ and GFR ≥60 ml/min/1.73 m^2^; the duration of follow-up time <12 months and ≥12 months.

The analysis was conducted using the Review Manager software (version 5.3). A value of *p* < 0.05 was considered statistically significant for all analyses.

## Results

The search identified 10 studies including 18,362 (9,173 patients in the interventional group and 9,189 patients in the control group) with a median follow-up of 10.5 months (range from 2 to 35 months). They were male predominant (65.4%, 12,016/18,362), with a mean age of 67 years old. All the studies were prospective double-blinded randomized controlled trials. The study size ranged from 118 to 8,399 with a median number of 439 patients. Overall, 6.4% (59/9,173) of the patients in the interventional group and 3.4% of the patients (31/9,189) in the control group were lost to the study follow-up. Among the 10 studies, one study (United Kingdom HARP-III, 414 patients, 15 of them reported heart failure) assessed CKD patients with an eGFR of 20–60 ml/min/1.73 m^2^, one study [Post-STEMI (Rezq et al., 2021), 200 patients] assessed patients after ST-segment elevation myocardial infarction. Five studies [54.9%, 10,085/18,362, PARADIGM (McMurray et al., 2014), PIONEER (Velazquez et al., 2019), EVALUATE (Desai et al., 2019), PRIME (Kang et al., 2019), and PARALLEL (Tsutsui et al., 2021)] assessed patients with HFrEF, while the other three [41.7%, 7,663/18,362, PARAMOUNT (Solomon et al., 2012), PARAGON (Solomon et al., 2019), and PARALLAX (Wachter et al., 2020)] assessed patients with HFpEF. Seven studies reported the stratified baseline eGFR level, the majorities of patients (60.5%, 9,198/15,214) had an eGFR ≥60 ml/min/1.73 m^2^ at baseline, while 39.5% (6,016/15,214) of the patients had a baseline eGFR <60 ml/min/1.73 m^2^. All studies except EVALUATE reported prior diabetes as the baseline characteristic, 37.3% (6,669/17,898) of the patients had a history of diabetes.

In the interventional group, the sacubitril/valsartan dosage was the same at 200 mg twice daily for nine studies except for the Post-STEMI, in which the dosage was 100 mg twice daily. Different RAASi were used in the control group, including valsartan 320 mg daily (28.3%, 2,599/9,189, PARAMOUNT, PARAGON, and PRIME), enalapril 20 mg daily (54.4%, 4,998/9,189, PARADIGM, PIONEER, EVALUATE, and PARALLEL), ramipril 10 mg daily (1.1%, 100/9,189, Post-STEMI), and irbesartan 300 mg once daily (2.3%, 207/9,189, United Kingdom HARP-III). The remaining 13.9% of the patients in the PARALLAX study were randomized to the control group receiving either enalapril 20 mg daily, valsartan 320 mg daily, or no RAASi (Table 1).

**TABLE 1:** Baseline characteristics of included trials.

Trial	2012 PARAMOUNT (*n* = 301)		2014 PARADIGM (*n* = 8,399)		2018 UK HARP-III (*n* = 414)		2018 PIONEER (*n* = 881)		2019 PARAGON (*n* = 4,796)		2019 EVALUATE (*n* = 464)		2019 PRIME (*n* = 118)		2020 PARALLAX** (*n* = 2,566)		2021 Post-STEMI (*n* = 200)		2021 PARALLEL (*n* = 223)	
Sac/val/RAASi	Sac/val *n* = 149	Val *n* = 152	Sac/val *n* = 4,187	Ena *n* = 4,212	Sac/val *n* = 207	Irb *n* = 207	Sac/val *n* = 440	Ena *n* = 441	Sac/val *n* = 2,407	Val *n* = 2,389	Sac/val *n* = 231	Ena *n* = 233	Sac/val *n* = 60	Val *n* = 58	Sac/val *n* = 1,281	IMT *n* = 1,285	Sac/val *n* = 100	Ram *n *= 100	Sac/val *n* = 111	Ena *n* = 112
Trial drug target dosage	200 mg twice daily	160 mg twice daily	200 mg twice daily	10 mg twice daily	200 mg twice daily	300 mg once daily	200 mg twice daily	10 mg twice daily	200 mg twice daily	160 mg twice daily	200 mg twice daily	10 mg twice daily	200 mg twice daily	160 mg twice daily	200 mg twice daily	NA	100 mg twice daily	5 mg twice daily	200 mg twice daily	10 mg twice daily
Female number (n)*	85	85	879	953	59	57	113	133	1,241	1,238	61	48	26	20	641	655	86	88	96	96
SBP (mmHg)	137	136	122	121	146	146	120	120	130.5	130.6	131	130	118.5	117	133	134	97.4	95.6	123.6	121.2
HR (bpm)	69	70	72	73	NA	NA	82	81	71	70	68	68	73.5	73.5	NA	NA	91.5	91.0	73.9	72.3
NT-pro BNP (pg/ml)	876	1,004	1,890	1,928	NA	NA	3,299	2,939	992	998	771	759	NA	NA	NA	NA	NA	NA	685.8	818.6
NT-pro BNP (pg/ml)^†^	794	870	1,681	1,594	254.5	250.9	2,972	2,536	NA	NA	575	575	NA	NA	786	760	NA	NA	NA	NA
EF (%)	58	58	29.6	29.4	NA	NA	24	25	57.6	57.5	34	33	34.3	33.3	57	56	NA	NA	28.6	27.7
Scr (mg/dl)	NA	NA	1.13	1.02	NA	NA	1.29	1.27	1.1	1.1	NA	NA	1.00	0.98	NA	NA	1.15	1.25	NA	NA
eGFR (ml/min/1.73m^2^)	66.5	64.3	NA	NA	35.4	35.5	59.1	59.1	63	62	70	69	NA	NA	NA	NA	NA	NA	58.3	57.6
GFR ≥ 60 (n)*	93	83	2,854	2,800	0	0	310	321	1,243	1,211	NA	NA	NA	NA	NA	NA	100	96	43	43
GFR < 60 (n)*	56	69	1,333	1,412	207	207	130	120	1,164	1,177	NA	NA	NA	NA	NA	NA	0	4	68	69
UACR (mg/mmol)	2.47	2.1	1.9	1.9	75	71	NA	NA	NA	NA	NA	NA	NA	NA	NA	NA	NA	NA	NA	NA
UACR (mg/mmol)^‡^	2.4	2.1	1.2	1.2	52	56	NA	NA	NA	NA	NA	NA	NA	NA	NA	NA	NA	NA	NA	NA
Baseline RAASi treatment (%)	93	93	99.7	99.8	84	80	47.3	48.5	86.2	86.4	81	88	100	100	87	88	NA	NA	71/40^#^	69/43^#^
Prior diabetes (n)	61	53	1,451	1,456	81	83	79	89	1,046	1,016	NA	NA	19	17	500	540	40	34	52	52
Follow-up duration	36 weeks		27 months		12 months		8 weeks		35 months		12 weeks		12 months		24 weeks		6 months		33.9 months	

Note. *Data are presented as n.

**The data from PARALLAX trial was from https://clinicaltrials.gov/ct2/show/results/NCT03066804?view=results.

^†^Geometric means are presented for NT-pro BNP.

^‡^Medians are presented for UACR.

^#^Baseline ACEi/ARB treatment (%) are presented.

Data are presented as mean.

RAASi, renin–angiotensin–aldosterone system inhibitor; SBP, systolic blood pressure; HR, heart rate; NA, not available; NT-pro BNP, n-terminal pro-B-type natriuretic peptides; EF, ejection fraction; Scr, serum creatinine; eGFR, estimated glomerular filtration rate; UACR, urine albumin-to-creatinine ratio; Sac/val, sacubitril/valsartan; Val, valsartan; Ena, enalapril; Irb, irbesartan; Ram, ramipril; IMT, individualized medical therapy.

### Renal Outcomes

**FIGURE 2:**
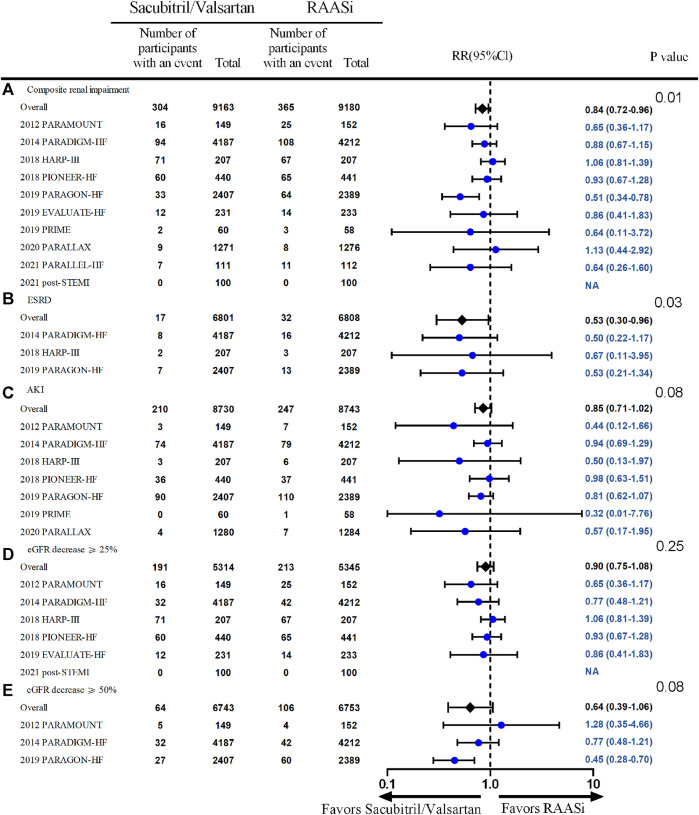
Effect of sacubitril/valsartan on renal outcome—the outcomes of composite renal outcome, ESRD, AKI, eGFR decrease ≥25%, and eGFR decrease ≥50% are presented as **(A,B,C,D,E)** respectively. Outcomes, above except eGFR decrease ≥50% **(E)**, were from fixed-effect meta-analysis. RAASi, renin–angiotensin–aldosterone system inhibitor; RR, relative ratio; AKI, acute kidney injury; ESRD, end-stage renal disease; eGFR, estimated glomerular filtration rate.

Compared with patients in the control group, patients treated with sacubitril/valsartan had lower incidence of composite renal impairment (10 studies, 18,362 patients, RR 0.84; 95% CI 0.72–0.96, *p* = 0.01; *I* ^2^ = 22%) and development of ESRD (3 studies, 13,609 patients, RR 0.53; 95% CI 0.30–0.96, *p* = 0.03; *I* ^*2*^ = 0%). Furthermore, there was no significant difference in the incidence of acute kidney injury (seven studies, 17,473 patients, RR 0.85; 95% CI 0.71–1.02, *p* = 0.08; *I* ^2^ = 0%), over 25% of reductions in the eGFR from baseline (six studies, 10,659 patients, RR 0.90; 95% CI 0.75–1.08, *p* = 0.25; *I* ^2^ = 0%) and over 50% of reductions in the eGFR from baseline (three studies, 13,496 patients, RR 0.64; 95% CI 0.39–1.06, *p* = 0.08; *I* ^2^ = 51%) between these two groups during a median follow-up time of 10.5 months (Figure 2). The definitions of worsening renal function (WRF) in each study are listed in Supplementary Table S2.

**FIGURE 3:**
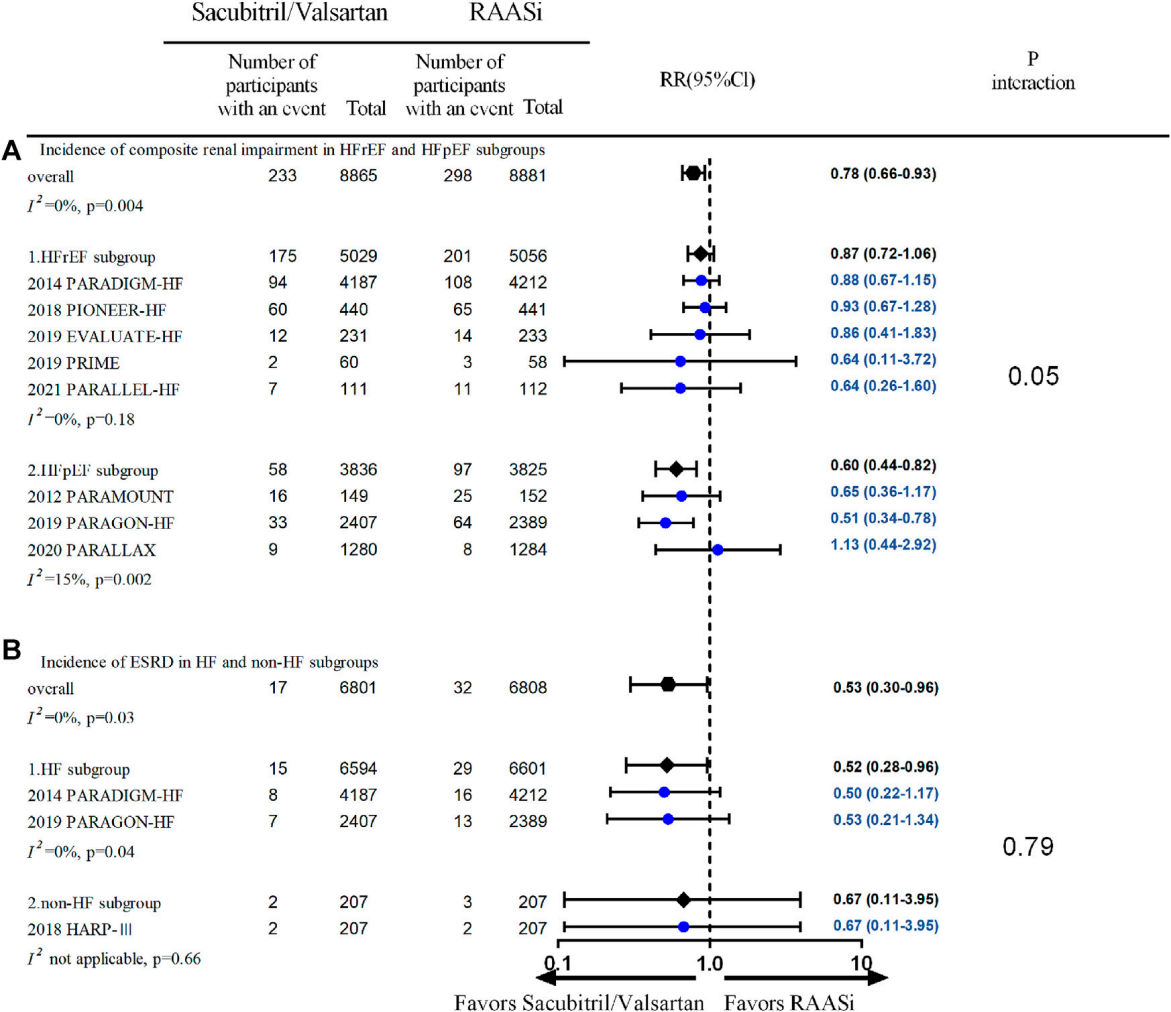
**(A)** Incidence of composite renal impairment in HFrEF and HFpEF subgroups—outcomes were from fixed-effect meta-analysis. RAASi, renin–angiotensin–aldosterone system inhibitor; RR, relative ratio; HFrEF, heart failure with reduced ejection fraction; HFpEF, heart failure with preserved ejection fraction. **(B)** Incidence of ESRD in HF and non-HF subgroups—outcomes were from fixed-effect meta-analysis. ESRD, end-stage renal disease; RR, relative ratio; HFrEF, heart failure with reduced ejection fraction; HFpEF, heart failure with preserved ejection fraction; RAASi, renin–angiotensin–aldosterone system inhibitor.

Subgroup analysis showed that the lower incidence of composite renal outcomes was seen in patients with HFpEF receiving sacubitril/valsartan treatment (three studies, 7,661 patients, RR 0.60; 95% CI 0.44–0.82, *p* = 0.002; *I* ^2^ = 15%), but not in patients with HFrEF (five studies, 10,085 patients, RR 0.87; 95% CI 0.72–1.06, *p* = 0.18; *I* ^2^ = 0%) (Figure 3A); the lower incidence of ESRD was seen in patients with HF receiving sacubitril/valsartan treatment (two studies, 13,195 patients, RR 0.52; 95% CI 0.28–0.96, *p* = 0.04; *I* ^2^ = 0%), but not in patients without HF (one study, 414 patients, RR 0.67; 95% CI 0.11–3.95, *p* = 0.66; *I* ^2^ not applicable) (Figure 3B). Further subgroup analysis showed no significant difference between patients with baseline eGFR <60 ml/min/1.73 m^2^ and those with baseline eGFR ≥60 ml/min/1.73 m^2^, follow-up duration <12 months and ≥12 months in the incidence of composite renal impairment, AKI, and development of ESRD between the two treatment strategies.

**FIGURE 4:**
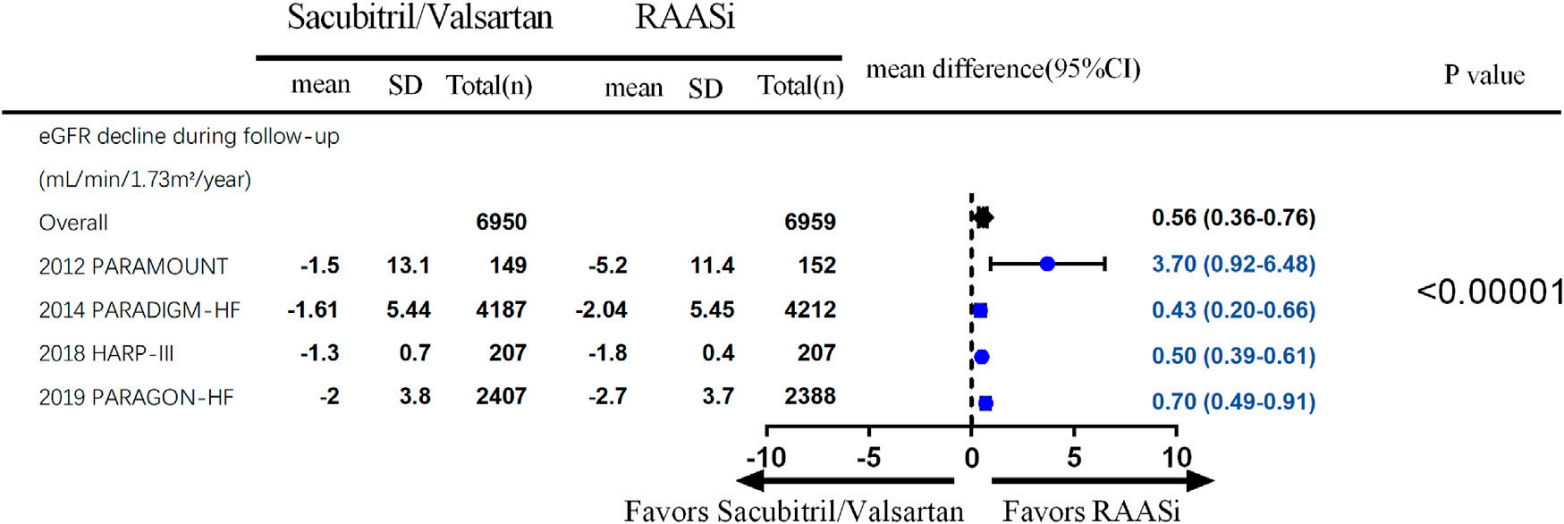
eGFR decline during the follow up-outcomes were from fixed-effect meta-analysis. RAASi, renin–angiotensin–aldosterone system inhibitor; eGFR, estimated glomerular filtration rate.

**FIGURE 5:**
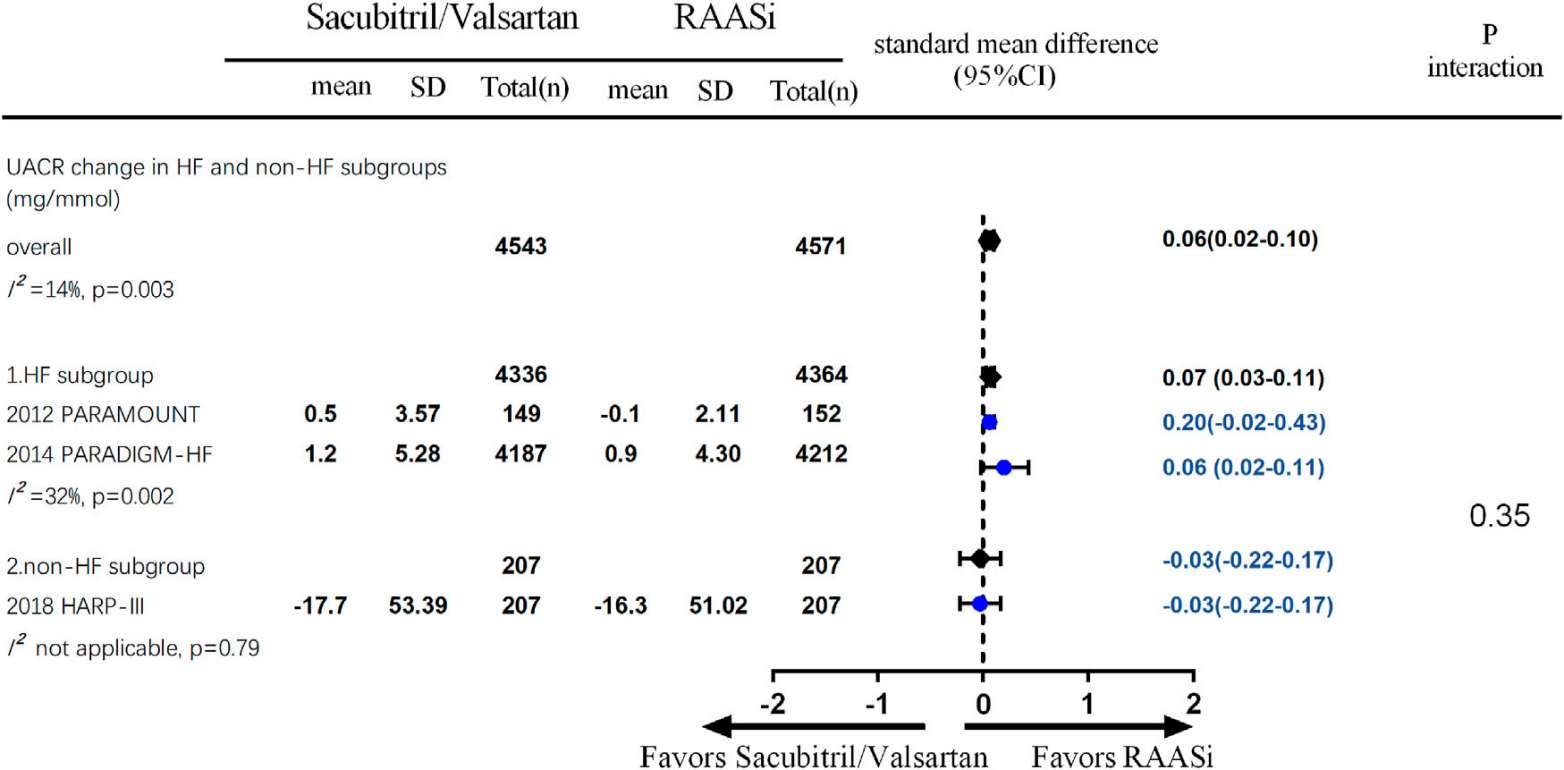
UACR change during the follow-up—outcomes were from random-effect meta-analysis. RAASi, renin–angiotensin–aldosterone system inhibitor; UACR, urine albumin-to-creatinine ratio.

Four studies (PARAMOUNT, PARADIGM, United Kingdom HARP-III, and PARAGON) reported changes in eGFR, and three studies reported UACR change during follow-up. Fewer decline in kidney function were seen in patients in the sacubitril/valsartan group compared with the RAASi group (four studies, 13,909 patients, WMD 0.53; 95% CI 0.44–0.62, *p* < 0.001; *I* ^2^ = 65%) (Figure 4). A smaller increase in UACR was seen in patients in the sacubitril/valsartan group than in the RAASi group during follow-up (three studies, 9,114 patients, SMD 0.06; 95% CI 0.02–0.10, *p* = 0.003; *I* ^2^ = 14%) between the two groups. Further subgroup analysis demonstrated an increase in UACR in patients with heart failure (PARAMOUNT and PARADIGM, 8,700 patients, SMD 0.07; 95% CI 0.03–0.11, *p* = 0.002; *I* ^2^ = 32%), rather than the non-heart failure group (chronic kidney disease group, United Kingdom HARP-III, 414 patients, SMD −0.03; 95% CI −0.22 to 0.17, *p* = 0.79) (*p* = 0.35 for interaction) (Figure 5).

### Compliance and Adverse Events

**FIGURE 6:**
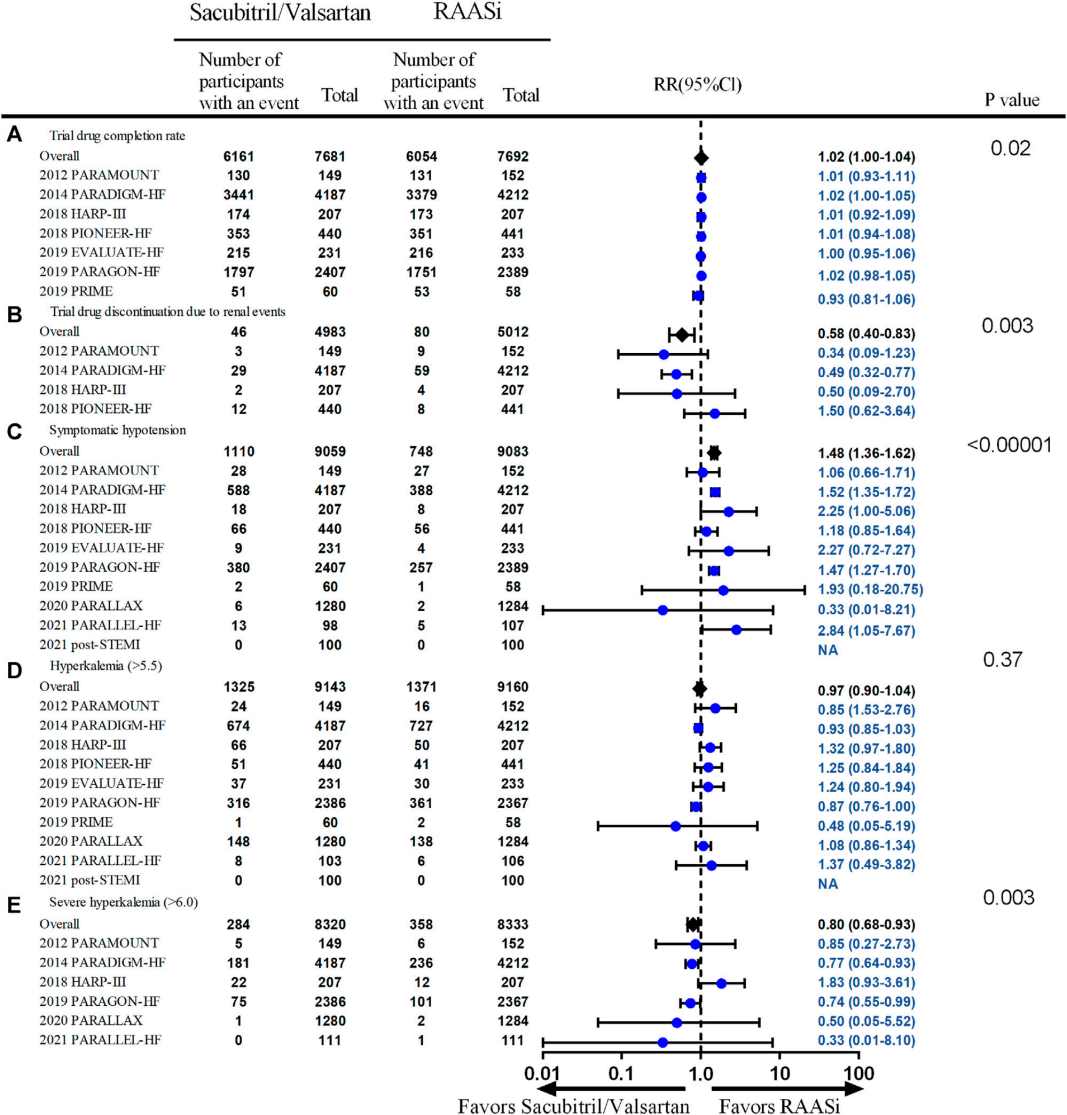
Compliance and safety outcomes—outcomes of trial drug completion rate, trial drug discontinuation due to renal events, systematic hypotension, hyperkalemia, and severe hyperkalemia. Outcomes were from fixed-effect meta-analysis. RAASi, renin–angiotensin–aldosterone system inhibitor; RR, relative ratio.

Overall, patients in both groups were compliant with the study drugs (79.7% of the patients achieved the target dose in the interventional group and 78.5% patients in the control group). Compliance with sacubitril/valsartan appeared to be better than RAASi (seven studies, 15,373 patients, RR 1.02; 95% CI 1.00–1.04, *p* = 0.02; *I* ^2^ = 0%), and less patients discontinued to take the medication due to renal events (four studies, 9,995 patients, RR 0.58; 95% CI 0.40–0.83, *p* = 0.003; *I* ^2^ = 47%). In view of adverse events, sacubitril/valsartan was associated with an increased risk of symptomatic hypotension (10 studies, 18,142 patients, RR 1.48; 95% CI 1.36–1.62, *p* < 0.00001; *I* ^*2*^ = 0%), but no differences in incidence of total severe adverse events. No differences in angioedema (10 studies, 18,360 patients, RR 1.59; 95% CI 0.95–2.64, *p* = 0.08, *I* ^2^ = 31%), cough (4 studies, 16,007 patients, RR 0.94; 95% CI 0.63–1.39, *p* = 0.75; *I* ^2^ = 81%), and hyperkalemia (serum potassium >5.5 mmol/L) (10 studies, 18,303 patients, RR 0.97; 95% CI 0.90–1.04, *p* = 0.37, *I* ^2^ = 41%) were seen between sacubitril/valsartan and RAASi treatment, but the incidence of severe hyperkalemia (serum potassium >6.0 mmol/L) was lower in the intervention group (six studies, 16,653 patients, RR 0.80; 95% CI 0.68–0.93%, *p* = 0.003, *I* ^2^ = 25%) (Figure 6).

Subgroup analysis showed that there were no differences in the incidence of hyperkalemia or severe hyperkalemia according to different heart failure types between sacubitril/valsartan and RAASi treatment. Further subgroup analysis showed no significant difference between patients with baseline GFR <60 ml/min/1.73 m^2^ and those with baseline GFR ≥60 ml/min/1.73 m^2^ in the incidence of hyperkalemia (serum potassium >5.5 mmol/L); not enough data was available in terms of severe hyperkalemia.

### Quality of Trials

The risk of bias for each included randomized controlled trial is shown in Supplementary Figures S1 and S2. Each trial generated the allocation sequence randomly. Of the included trials, including PARADIGM, PIONEER, EVALUATE, PARALLEL, and Post-STEMI, 5/10 did not describe detailed methods for concealment allocation. The blinding of participants and personnel was judged to be low for all included trials except the PARALLEL trial. The blinding of outcome assessors was judged to be low for each trial. There was a low risk from incomplete outcome reporting in the included trials. Low risk for selective reporting was judged for each included trial. Intention-to-treat analysis was used in all trials except four trials, which were PARAMOUNT, EVALUATE, PRIME, and PARALLEL.

## Discussions

There is increasing evidence on the cardiovascular benefits of sacubitril/valsartan in patients with heart failure with both reduced and preserved ejection fraction (Solomon et al., 2020). However, its effect on kidney function remains inconsistent. In the secondary analysis of the PARAMOUNT trial (Voors et al., 2015), LCZ696 therapy was associated with preservation of eGFR compared with valsartan therapy in patients with HFpEF for 36 weeks, but with an increase in UACR. In the secondary analysis of the PARADIGM-HF trial, sacubitril/valsartan led to a slower decrease rate in the eGFR compared with enalapril alone, despite causing a modest increase in UACR (Damman et al., 2018) in patients with HFrEF with baseline mean eGFR of 70 ml/min/1.73 m^2^ followed up to 27 months. In the second analysis of PARAGON-HF trail, sacubitril/valsartan reduced the risk of renal events and slowed the decline in eGFR in patients with HFpEF, in comparison with valsartan (Mc Causland et al., 2020), while this renal protective effect seemed to disappear in patients with more advanced chronic kidney disease (mean eGFR of 34 ml/min/1.73 m^2^) but comparable UACR decrease in the United Kingdom HARP-III trial (Haynes et al., 2018).

In this systematic review, we assessed the effect of sacubitril/valsartan on both heart failure and non-heart failure renal outcomes of patients. To the best of our knowledge, it is the latest meta-analysis to enroll the most patients to compare sacubitril/valsartan treatment with RAASi on renal efficacy and safety. As to the efficacy, we found that the use of sacubitril/valsartan was associated with lower incidence of composite renal impairment in patients with heart failure and preserved ejection fraction, lower incidence of ESRD development in patients with both HFrEF and HFpEF, fewer eGFR decrease in patients with both heart failure or non-heart failure, but higher albuminuria in patients with heart failure (both HFrEF and HFpEF). As to the compliance and safety, although a higher incidence of symptomatic hypotension was seen in the sacubitril/valsartan group, compliance with sacubitril/valsartan appeared to be better than RAASi, including higher trial drug completion rate, lower drug discontinuation due to renal events, and incidence of severe hyperkalemia. Further baseline eGFR subgroup analysis of renal efficacy and safety did not find differences between eGFR <60 ml/min/1.73 m^2^ and eGFR ≥60 ml/min/1.73 m^2^ and neither did the subgroup analysis of follow-up duration. The renal function preservation of sacubitril/valsartan was consistent with a previous meta-analysis that enrolled different RCTs (Spannella et al., 2020), but the difference between the two meta-analyses will be discussed later.

It is well known that the progression of CKD is mediated by glomerular hyperfiltration, oxidative stress, chronic inflammation, and fibrosis. The blockade of the RAAS has been demonstrated to be the most effective strategy to delay the deterioration process in the past two decades. However, the side effect and limited efficacy of the RAASi treatment prompted new treatment strategies. Neprilysin, which is the richest expressed in the brush border of the renal proximal tubular cell, is a key enzyme to degrade natriuretic peptides. The inhibition of neprilysin results in vasodilatation, which could ameliorate glomerular hypertension. The dual inhibition of neprilysin and RAAS has been demonstrated to achieve better renal protection beyond RAASi alone in animal studies. The possible mechanism is not only limited to the glomerular hemodynamic amelioration but also to the reduction of oxidative stress, inflammation, and fibrosis. Our meta-analysis demonstrated that sacubitril/valsartan treatment showed superior renal protection compared with RAASi irrespective of the baseline eGFR level, while subgroup analysis results showed that this effect was more obvious in heart failure patients. The mechanism behind the results could be that the neprilysin levels are elevated in HF patients, and the additional inhibition of neprilysin could delay eGFR decline on the basis of sufficient RAAS inhibition (Jing et al., 2017).

The subgroup analysis of prior diabetes was available only in the United Kingdom HARP-III trial (Haynes et al., 2018) and a *post-hoc* analysis of the PARADIGM trial in terms of renal outcomes (Packer et al., 2018). In the United Kingdom HARP-III trial, a nonstatistically significant superior eGFR preservation was seen in the sacubitril/valsartan group compared with the irbesartan group [difference in means 0.1 ml/min/1.73 m^2^ (95% CI −2.0 to 2.2, *p* = 0.76) at 12 months after randomization] in patients with prior diabetes. In the secondary analysis of PARADIGM, patients treated with sacubitril/valsartan had a slower rate of decline in eGFR (−1.3 vs. −1.8 ml/min/1.73 m^2^ per year; *p* < 0.0001), and the magnitude of the benefit was larger in patients with versus those without diabetes [difference 0.6 ml/min per 1.73 m^2^ per year (95% CI 0.4–0.8) in patients with vs. 0.3 ml/min/1.73 m^2^ per year (95%CI 0.2–0.5) in those without diabetes, *p* = 0.038]. This net benefit for diabetic patients should be verified in further clinical trials.

Although renal protection was demonstrated in this meta-analysis, albuminuria excretion seemed to increase in HF patients with sacubitril/valsartan treatment, which was not parallel to the preservation of renal function. The mechanism of UACR increase could not be analyzed thoroughly due to the limited reported data from only three RCTs. A previous study has noticed that the increased excretion may be due to a higher concentration of atrial natriuretic peptides (Vervoort et al., 2002). As we know, the signature response to activation of natriuretic peptides is an increase in their second messenger cyclic guanosine monophosphate (cGMP). Data from the appendix of the PARADIGM trial demonstrated that urinary cGMP level did not change much after randomization in the sacubitril/valsartan group but fell to approximately 60% of the baseline level in the enalapril group (McMurray et al., 2014). The increase in cGMP could inhibit tubuloglomerular feedback both dependent and independent of changes in proximal tubular sodium reabsorption (Lessa et al., 2012), increased albuminuria may be the result of increased susceptibility to and availability of cGMP in combination with higher natriuretic peptide levels, which enhances podocyte permeability (Störk, 2018). In contrast, a previous animal study showed that a combination of neprilysin and ACEI could improve blood pressure and proteinuria, and delay renal function decline (Taal et al., 2001). Further studies are urgently needed to explain the exact mechanism of the UACR change with sacubitril/valsartan treatment. It is noteworthy that the EMPAREG-OUTCOME trial (Packer et al., 2021) demonstrated the beneficial effects of the SGLT2 inhibitor empagliflozin on both eGFR and urinary albumin–creatinine ratio. The dual inhibition of both neprilysin and SGLT2 may be a promising strategy to preserve kidney function and ameliorate UACR in patients with heart failure.

Our study demonstrated better compliance with sacubitril/valsartan than RAASi. There was a 42% decreased risk of drug discontinuation due to renal events and a 20% decreased risk of severe hyperkalemia in patients receiving sacubitril/valsartan, suggesting that sacubitril/valsartan is well-tolerated in patients with or without heart failure or chronic kidney disease. The incidence of hyperkalemia was not different between subgroups of baseline eGFR <60 ml/min/1.73 m^2^ and eGFR ≥60 ml/min/1.73 m^2^. Sacubitril/valsartan, however, was associated with a 49% increased risk of symptomatic hypotension compared with RAASi treatment. A higher risk of hypotension is probably due to sacubitril, which curtails the degradation of natriuretic peptides with vasodilatory properties (Jhund and McMurray, 2016). Our meta-analysis results revealed a safe profile of sacubitril/valsartan treatment on the premise of strict blood pressure monitoring.

There are similarities between previous (Spannella et al., 2020) and current meta-analyses. We both came to the same conclusion that sacubitril/valsartan has superior renal protection compared with RAASi. Meanwhile, there are significant differences between them. First, there were more latest RCTs enrolled in our meta-analysis, including the Post-STEMI, PARALLEL, and PARALLAX trials. The sample size in our analysis is bigger than theirs (18,362 vs. 16,456). In our meta-analysis, three RCTs enrolled in the previous one were not included, one of them had unclear renal outcomes termed “severe renal impairment,” while the other two targeted patients with hypertension and uncontrolled hypertension, which could impact the renal outcomes directly. Second, we focused on different subgroup analyses. The purpose of our analysis was to find out the patients who could benefit most, so we put emphasis on the baseline eGFR and follow-up duration subgroups to explore the admittance criterion and tolerance time with acute kidney injury. Although no significant differences were observed in our analysis, more data from further trials are pivotal to verify this problem. Third, we evaluated the adverse events especially the incidence of hyperkalemia, which was important concerning the treatment of RAASi. Our result showed a safe profile of sacubitril/valsartan treatment irrespective of the baseline eGFR level. However, the conclusion should be drawn cautiously due to the insufficient data.

Our study has several limitations. First, the longest follow-up time among included trials was 35 months, so the long-term effect of sacubitril/valsartan on the renal outcomes cannot be determined due to lack of the long-term data. Second, there is heterogeneity in the study design, inclusion and exclusion criteria, as well as outcome assessment between the included studies, for example, the inclusion of a run-in phase that helped to stabilize some patients or excluded those with acute renal decompensation. As a result, the selection of low-risk patients may occur. The way we tried to handle this issue was to conduct multiple subgroup analyses. Third, there are some variations in the patients’ demographic and clinical characteristics among the included studies. Though we conducted multiple subgroup analysis to address this issue, the limited number of trials for assessment of some outcomes necessitates further RCTs to assess such outcomes. Last, although the HARP-III population is patients with preexisting chronic kidney disease, about 3.6% (15/414) of them self-reported history of heart failure; however, the average baseline NT pro-BNP level was lower than the inclusion criteria of the PARADIGM and PARAGON trials (254.5 ng/L in the sacubitril/valsartan group and 250.9 ng/L in the irbesartan group, respectively) (UK HARP-III Collaborative Group, 2017). Since NT pro-BNP is cleared by the kidney, insufficient renal function will interfere with and limit the diagnostic value of NT pro-BNP for heart failure (Cataliotti et al., 2001). Due to the lack of detailed information separating heart failure and non-heart failure patients in this study, we were unable to assess the renal effects on the heart failure cohort in this study. Since only 2.3% of the patients included (United Kingdom HARP-III population) did not have heart failure, 1.1% of patients included (Post-STEMI) did not report baseline heart function. The conclusions drawn from this review are a reflection of renal outcomes mainly in HF patients.

## Conclusion

Current evidence from prospective randomized controlled studies suggest that the use of sacubitril/valsartan, compared with RAASi, was associated with lower incidence of composite renal impairment in patients with HFpEF, lower incidence of ESRD development in patients with HF, but higher microalbuminuria in patients with heart failure (both HFrEF and HFpEF). The compliance and safety profile was superior in sacubitril/valsartan treatment patients except for the symptomatic hypotension. Due to the existence of heterogeneity of the population and follow-up duration, more high-quality randomized controlled trials are needed to assess this issue, especially focusing on the subgroup analysis of different baseline CKD stages and prior diabetes history.

## Data Availability

The original contributions presented in the study are included in the article/Supplementary Material, further inquiries can be directed to the corresponding authors.

## Abbreviations

ACEI, angiotensin-converting enzyme inhibitor; AKI, acute kidney injury; ESRD, end-stage renal disease; CKD, chronic kidney disease; CI, confidence interval; eGFR, estimated glomerular filtration rate; HF, heart failure; HFpEF, heart failure and preserved ejection fraction; HFrEF, heart failure and reduced ejection fraction; RR, relative ratio; RAASi, renin–angiotensin–aldosterone system inhibitor; SMD, standard mean difference; UACR, urine albumin-to-creatinine ratio; WMD, weighted mean difference.
