# Supplementary material for: Effect of Angiotensin–Neprilysin Versus Renin–Angiotensin System Inhibition on Renal Outcomes: A Systematic Review and Meta-Analysis
Source: Front Pharmacol. 2021 Nov 19;12:604017. doi: 10.3389/fphar.2021.604017 (PMC8640344; doi:10.3389/fphar.2021.604017)
Supplement: Supplementary file 2 [file DataSheet1.docx]

**Appendix**

**Table S1. Search strategy**

***EMBAS via Ovid***

1. 'sacubitril plus valsartan'/exp
2. Entresto
3. 'lcz 696'
4. 'sacubitril valsartan'
5. neprilysin AND inhibito*
6. 'enkephalinase inhibitor'/exp
7. Omapatrilat
8. or/1-7
9. 'randomized controlled trial'/exp
10. 'randomized controlled trial'
11. 'clinical trial'/exp
12. 'double blind procedure'/exp
13. 'single blind procedure'/exp
14. 'random allocation'
15. random$: ab, ti OR placebo$:ab, ti
16. or/9-15
17. 'heart failure'/exp
18. heart* NEAR/2 decomp*
19. (heart* OR cardiac* OR myocard*) NEAR/2 (fail* OR insuff*)
20. or/17-19
21. 8 AND 16 AND 20

***MEDLINE via Ovid***

1. Entresto.mp.
2. LCZ 696.mp.
3. sacubitril valsartan.mp.
4. neprilysin inhibito*.mp.
5. omapatrilat.mp.
6. or/1-5
7. exp Heart Failure/
8. ((heart* or cardiac* or myocard*) adj2 (fail* or insuff*)).tw.
9. (heart* adj2 decomp*).tw.
10. or/7-9
11. exp Controlled Clinical Trial/
12. exp Randomized Controlled Trials as Topic/
13. exp Random Allocation/
14. exp Double-Blind Method/
15. exp Single-Blind Method/
16. Clinical Trial.tw.
17. Placebo.tw.
18. (clinic$3 adj trial$2). ab, ti.
19. (clin$ adj25 trial$).mp.
20. or/11-19
21. 6 and 10 and 20

***Cochrane library***

1. entresto
2. lcz 696

3 neprilysin inhibit*

4 sacubitril valsartan

5 or/1-4

6 MeSH descriptor: [Heart Failure] explode all trees

7 (heart* or cardiac* or myocard*) near2 (fail* or insuff*)

8 heart* near2 decomp*

9 or/6-8

10 5 and 9

**Table S2. The definitions of worsening renal function (WRF) in each trial**

| Trial | The definitions of worsening renal function |
| --- | --- |
| 2012 PARAMOUNT | An serum creatinine increase of >0.3mg/dL and/or >25% between two time-points |
| 2014 PARADIGM | Reaching end-stage renal disease or a decrease in the eGFR of at least 50% or a decrease of more than 30 ml/min/1.73 m^2^ from randomization to less than 60 ml/min/1.73 m^2^ |
| 2018 UK HARP-III | Decline of ≥25% in eGFR |
| 2018 PIONEER | An increase in the serum creatinine concentration of ≥0.5 mg/dL [≥44 μmol/L] and a decrease in the eGFR of ≥25% |
| 2019 EVALUATE | Decrease in eGFR of ≥35% or increase in serum creatinine of ≥ 0.5 mg/dL from baseline and decrease in eGFR of ≥25% from baseline. |
| 2019 PARAGON | Decrease in the eGFR of ≥50%, development of end-stage renal disease, or death due to renal failure |
| 2019 PRIME | Acute renal failure or serum creatinine ≥2.5 mg/dL |
| 2020 PARALLAX | Acute kidney injury or renal failure or renal impairment or renal injury |
| 2021 PARALLEL | Serum creatinine ≥2.0 mg/dL |
| 2021 Post-STEMI | An increase in the serum creatinine concentration of ≥ 0.5 mg/dL [≥ 44 μmol/L] and/or a decrease in the eGFR of ≥ 25% |


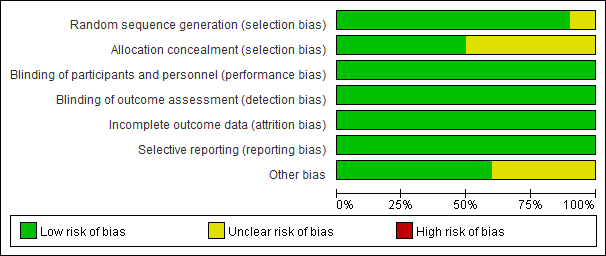


**Supplementary Figure 1. Risk of bias graph: review authors' judgements about each risk of bias item presented as percentages across all included studies.**


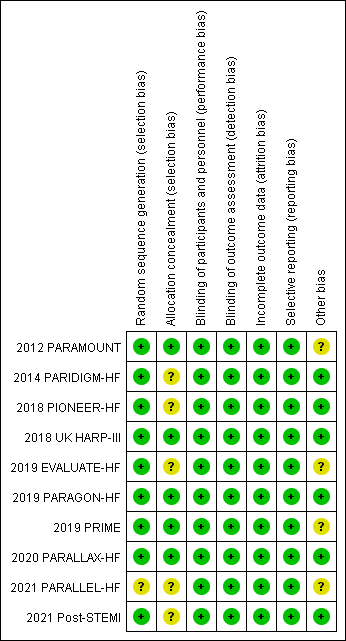


**Supplementary Figure 2. Risk of bias summary: review authors' judgements about each risk of bias item for each included study.**
